# Botulinum as a Toxin for Treating Post-herpetic Neuralgia

**Published:** 2017-05

**Authors:** Xu-Dong DING, Jing ZHONG, Yan-Ping LIU, Hua-Xian CHEN

**Affiliations:** 1. Dept. of Rehabilitation Medicine, Xiangyang No.1 People’s Hospital, Hubei University of Medicine, Xiangyang, Hubei, China; 2. Dept. of Ultrasound Imaging, Xiangyang No.1 People’s Hospital, Hubei University of Medicine, Xiangyang, Hubei, China

**Keywords:** Postherpetic neuralgia, Botulinum a toxin, Treatment

## Abstract

**Background:**

We aimed to study the curative effects of botulinum A toxin (BTX-A) on the treatment of post-herpetic neuralgia (PNH).

**Methods:**

We enrolled 58 PNH patients and treated them with hypodermic injection of BTX-A in Xiangyang No.1 People’s Hospital, Hubei University of Medicine, Hubei, China. We measured and compared the Visual Analog Score (VAS), Neuropathy Pain Scale (NPS), Quality of Life Scale (SF-36) score, PNH seizure severity degree, seizure duration, frequency of attack and the use of painkillers before and after treatment. We used SPSS13 software package for statistical analysis. Values were expressed by mean± standard deviation. *P*<0.05 indicated a significant difference and P<0.01 indicated an obvious significant difference.

**Results:**

Attack frequency, attack duration and attack severity were all significantly lower after treatment (*P*<0.01). The use of painkillers reduced after treatment (*P*<0.01) and we observed very few adverse reactions associated with BTX-A injection.

**Conclusion:**

The use of BTX-A for treating post-herpetic neuralgia produced very promising results with very few adverse reactions. BTX-A can be considered as a valid approach in the treatment of PNH, especially in patients that do not respond well to painkillers.

## Introduction

Herpes zoster and post-herpetic neuralgia (PNH) result from reactivation of the varicella-zoster virus (VZV). Patients usually suffer from painful and itchy rash. The incidence of herpes zoster increases sharply with advancing age, and middle-aged individuals, especially the elderly are more inclined to suffer from this illness.

If herpes zoster is not treated in a timely manner, then patient may develop post-herpetic neuralgia (PHN). PHN pain can persist up to 1 to 2 years after resolution of the rash and can be highly debilitating (1). Patients suffering long-term pain have poor quality of life, limited ability to work and are susceptible to develop depression. Usually, common drug treatment produces dismal outcome with poor effects with obvious side effects (2). Botulinum A toxin (BTX-A) has been beneficial for patients suffering from PHN pain (1–4). We investigated the effects of BTX-A subcutaneous injection on 58 patients suffering from PHN pain.

## Materials and Methods

### Research Objects

Fifty-eight PNH patients were enrolled in this study. Patients did not respond well to drugs such as carbamazepine and phenytoin sodium. All patients were followed up for 6 months. There were 32 males and 26 females, aged from 34 to 79 yr (average=52.3 yr), and the course of disease was 4 to 15 months (average=9.5 months). Thirty-five patients needed to take painkillers for their PNH attacks. Patients with myasthenia gravis, Eaton-Lambert syndrome, motor neuron disease, allergic constitution, serious organs dysfunction, severe cognitive impairment, mental disorders, asthma history, fever and infectious diseases were excluded from this study. In addition, pregnant women and those who took medication for aggravated transmission disorders of neuromuscular junction, one week prior to this study were also excluded.

This study was approved by Ethics Committee of our hospital and all patients signed informed consent forms. All patients were subjected to blood routine, urine routine, liver and kidney function, ECG and EEG tests before the treatment.

### Treatment methods and follow-up

Before the injection, we measured Visual Analog Score (VAS), Neuropathy Pain scale (NPS) and the Quality of Life Scale (SF-36) score in all patients. PNH seizure severity degree, seizure duration, the frequency of attacks and the use of painkillers were also recorded.

Lyophilized crystalline BTX-A was obtained from LanZhou Institute of Biological. Each vial of BTX-A contained 100 U and was preserved at 2 to 8 °C. It was diluted in saline (25 ug/ml) before injection. Five U of BTX-A was injected subcutaneously (total dose was generally 50 to 100 U). Two weeks after the injection, the abovementioned indexes were measured again. Measurements were repeated every month for 6 months through outpatient service or telephone interviews.

### Pain severity

VAS was used to measure pain severity. 0 represented no pain and 10 represented excruciating pain. 0 point referred to no pain, 1 to 3 indicated mild pain, 4 to 6 showed moderate pain, 7 to 9 indicated severe pain, and 10 showed the most intense pain.

### Evaluation criteria for curative effect

The onset situation, 3 months prior to treatment was taken as a basic level, and the pain situation was studied. Severity, seizure duration, the frequency of the attack and the use of painkillers were measured. Scale for NPS and SF-36 indexes was as follows:

- Markedly effective: improvement by 50% and over;
- Effective: improvement of 25% to 49%;
- Ineffective: improvement below 25%.

Total effective rate= markedly effective rate+ effective rate.

#### Statistical analysis

We used SPSS13 software package for statistical analysis. Values were expressed by mean± standard deviation. *P*<0.05 indicated a significant difference.

## Results

In 18 (31%) cases BTX-A treatment was effective and in 27 (46.6%) cases BTX-A treatment produced significant results. In 13 patients BTX-A treatment was ineffective (22.4%). Two weeks after treatment, the frequency of pain attack reduced significantly in all patients (*P*<0.01). Compared with 3 months before treatment, the pain severity (VAS score) was significantly lower (*P*<0.01). After treatment, the seizure duration was significantly shortened (*P*<0.05), NPS score was significantly reduced (*P*<0.01), and SF-36 score was significantly improved (*P*<0.01) (Table 1). Compared with before treatment, the quantity of painkillers consumed by patients was reduced significantly after BTX-A treatment (*P*<0.01) (Table 2).

**Table 1:** Pain seizure before and after the treatment

**Indicators**	**Before the Treatment**	**Two Months After the Treatment**	**One Month After the Treatment**	**Three Months After the Treatment**	**Six Months After the Treatment**
Seizure Frequency (time/ month)	15.7±2.6	9.3±2.2*	7.2±1.5*	5.5±1.7*	9.5±1.9*
VAS score	8.1±0.3	5.0±0.4*	1.6±0.2*	1.5±0.2*	4.5±0.4*
NPS score	81.3±5.6	56.2±7.1*	33.7±3.2*	29.2±3.7*	45.5±5.1*
Seizure duration(h/time)	22.8±4.2	17.5±3.2^#^	7.2±2.9^#^	5.4±2.8^#^	9.5±2.1^#^
SF-36 score	35.7±13.7	51.5±14.6	84.2±17.1*	85.3±17.5*	74.5±16.3*

Note: compared with those before the treatment,

# *P*<0.05

* *P*<0. 01

**Table 2:** Painkiller requirement before and after the treatment

**Indicators**	**Before the treatment**	**Two weeks after the treatment**	**One month after the treatment**	**Three months after the treatment**	**Six months after the treatment**
The Required Amount of Painkiller (the number of tablets/ month)	30.3±4.6	19.5±3.5*	9.4±2.2*	6.6±2.4*	15.4±2.3*
The Number of Cases Requiring Painkiller	35 (60.3%)	24 (41.3%)	15 (25.8%)	13 (22.4%)	17 (29.3%)

Note: Compared with those before the treatment

* *P*<0.01

Efficacy duration of BTX-A treatment was ranged from 7 to 10 days, and the peak value was around one month.

After BTX-A injection, 4 patients complained about pain around the injection area, but pain disappeared after one week with no treatment.

## Discussions

PHN is a neuralgia that persists after a Herpes zoster rash has cleared. Pain often occurs as spontaneous lightning and aggravates when diseased skin touches the clothes. In addition, physical activities, temperature changes and emotional suppression may also deteriorate patients’ condition.

BTX-A is a type of A toxin secreted by *Clostridium botulinum* that can interfere with neurotransmitters. BTX-A can be used clinically for treating muscle cramps, opisthotonos, brain paralysis, hypermyotonia and strabismus. The VAS score in an 82-year-old PNH patient was lowered from 10 to 1 after BTX-A treatment and the curative effect lasted for 52 days (3).

We demonstrated that BTX-A significantly improved PNH symptoms and decreased the attack frequency. The severity of attack and attack lasting time was reduced after BTX-A injection and the quantity of painkillers consumed by patients decreased markedly. Compared with before treatment, life quality score improved significantly (*P*<0.01), which further confirmed the effectiveness of BTX-A treatment for PNH. Pain levels in 29 patients with PNH fell after the injection of BTX-A, and the curative effect lasted for 2 months (4).

The mechanism involved in the curative effect of BTX-A is still unclear, but a few explanations have been offered (5–9): (i) BTX-A may act on the presynaptic membrane of neuromuscular junction, inhibiting the release of neurotransmitter acetylcholine and producing chemicals to act on eneurosis and muscle relaxing; (ii) BTX-A may act on the muscle spindle afferent fiber to reduce the activity of muscle spindle and regulate sensory counter; (iii) BTX-A may inhibit the release of neuropeptides and neurogenic inflammation and reduce the force of afferent nerve; (iv) BTX-A may spread backwards central nervous system, directly regulating the expression of P material and encephalin, directly inhibiting the activity of neurovascular system and influencing central pain modulating system to relieve pains.

## Conclusion

Using BTX-A for treating PNH generated promising and long lasting results with very few adverse reactions. We believe that BTX-A can be considered as a valid approach in the treatment of PNH, especially in patients that do not respond well to painkillers.

## Ethical considerations

Ethical issues (Including plagiarism, informed consent, misconduct, data fabrication and/or falsification, double publication and/or submission, redundancy, etc.) have been completely observed by the authors.
